# Injectable Hydrogels in Cardiovascular Tissue Engineering

**DOI:** 10.3390/polym16131878

**Published:** 2024-07-01

**Authors:** Raj Patel, Dhruvi Patel

**Affiliations:** 1 Banas Medical College and Research Institute, Palanpur 385001, India; rajpatel7875@gmail.com; 2 School of Civil and Environmental Engineering, Cornell University, Ithaca, NY 14850, USA

**Keywords:** hydrogel, cardiovascular, 3D printing, drug delivery, tissue engineering

## Abstract

Heart problems are quite prevalent worldwide. Cardiomyocytes and stem cells are two examples of the cells and supporting matrix that are used in the integrated process of cardiac tissue regeneration. The objective is to create innovative materials that can effectively replace or repair damaged cardiac muscle. One of the most effective and appealing 3D/4D scaffolds for creating an appropriate milieu for damaged tissue growth and healing is hydrogel. In order to successfully regenerate heart tissue, bioactive and biocompatible hydrogels are required to preserve cells in the infarcted region and to bid support for the restoration of myocardial wall stress, cell survival and function. Heart tissue engineering uses a variety of hydrogels, such as natural or synthetic polymeric hydrogels. This article provides a quick overview of the various hydrogel types employed in cardiac tissue engineering. Their benefits and drawbacks are discussed. Hydrogel-based techniques for heart regeneration are also addressed, along with their clinical application and future in cardiac tissue engineering.

## 1. Introduction

The human heart is an incredible example of natural engineering where the contraction of the cardiac muscle pumps are nutrients and oxygen rich blood which flows throughout the body. The heart muscle receives blood flow via the coronary arteries (CA). In a matter of minutes, cardiac cell death brought on by CA constriction or obstruction results in myocardial infarction (MI). This causes the heart’s function to fail [1,2]. Collagen-containing scar tissue eventually fills the infarcted region in order to bear the increased pressure during the reduction cycle (systole). The heart’s ability to function fail when the scar tissue thins, leading to congestive heart failure (CHF). The leading cause of death globally is still cardiovascular diseases (CVDs), which include coronary heart disease, rheumatic heart disease, congenital heart disease, myocardial infarction (MI), and stroke [3,4,5,6].

Treatment involves transporting cells directly into the heart to promote cardiac rejuvenation and results in the integration of cells into the native heart to reform its muscle. There are several clinical studies being conducted regarding the implantation of stem cells in the heart in which a buffer is a widely utilized delivery method [7,8]. Very low quantities of oxygen and nutrients are triggered by coronary artery stenosis. Apoptotic cytokines and cell-toxic reactive oxygen species (ROS) are released concurrently with phagocytosis. A given cell’s ability to survive is limited by each of these elements [9,10]. A technique known as reformative cardiac tissue engineering, in which cells are given inside a supportive matrix, such as a scaffold or hydrogel, may increase the engraftment rate. The supporting matrix creates an environment that shields cells from the severe conditions of the MI heart and minimizes cell loss after injection. Furthermore, it serves as a mechanical support upon which to lower the increased wall stress brought on by the loss of healthy cardiac muscle cells, improving heart function. Because biomolecules may be easily added to the matrix to create a cell-friendly environment for improved regeneration, this method is also adaptable [11,12,13].

The World Health Organization (WHO) projects that the sum of CVD deaths will rise to 23.3 million by 2030, in spite of the discovery of innovative treatments and the implementation of risk-reducing measures [14]. As there are currently no viable treatments to entirely reestablish function of cardiovascular organ following injury, there is a high demand for the development of reformative techniques like stem-cell-based treatments and in vitro tissue engineering due to the shortage of organ donors. Currently, a variety of biomaterials can be employed to sustain tissue function, repair damaged components, or even stimulate tissue remodeling or regeneration. Metals, metal alloys, and naturally occurring and synthesized polymers are examples of these biomaterials [15,16,17,18]. Scaffold patches and hydrogels are the two primary materials that are in practice for cardiac tissue engineering. The focus of this study is on hydrogels that have a polymer matrix achieved via crosslinking and high-water content, and that are also insoluble in water. The modification and advancement of hydrogel-based biomaterials is one area of interest in the arena of reformative medicine. These materials may exhibit enhanced mechanical functioning or they may be bioactive, releasing growth factors, medicines, proteins, and extracellular matrix (ECM) components [19,20]. This review article delivers an outline of hydrogel-based biomaterials currently explored in the fields of cardiovascular tissue engineering and reformative medicine.

## 2. Types of Polymeric Hydrogels

**Figure 1:**
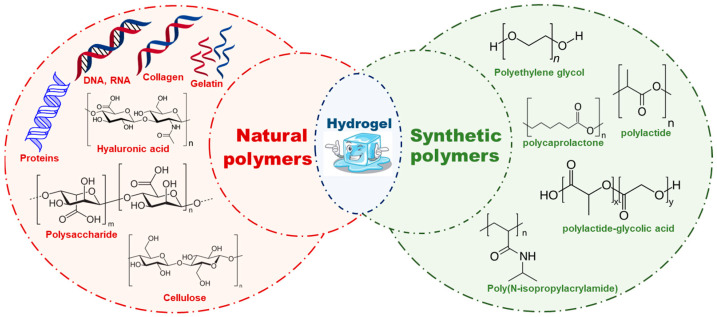
Injectable hydrogels showing their types, such as natural polymers and synthetic polymers with few examples and their chemical structures.

There are mainly two types of polymeric hydrogels are being used for cardiac tissue engineering i.e., natural and synthetic. Polymeric hydrogels are primarily used because of their stimuli responsiveness, softness, and viscoelasticity [21,22,23,24,25]. Some of the examples that have been discussed here are in Figure 1. Additionally, Table 1 contains detailed literature on the polymers used to formulate hydrogels; these have various properties that have been compiled with regard to its application in cardiac tissue engineering.

i. Natural Polymeric Hydrogels

Natural polymers, such as silk, wool, DNA, cellulose and proteins, arise in nature and are frequently water based. Collagen, gelatin, laminin, matrigel, hyaluronic acid, alginate, and chitosan are characteristic natural hydrogels, which, due to the identical structures in biological organisms, can reduce the prospect of immune response after in vivo implantation [26,27,28,29,30]. Some examples of natural polymeric hydrogels are discussed in this paper.

The majority of tissues’ mechanical strength is derived from collagen. The human body has a wide variety of collagen types, with type I collagen being the most widely distributed. Tissue engineering has made use of type I collagen because of its exceptional biocompatibility. Collagen can be dissolved in 0.3% glacial acetic acid solution to synthesize type I collagen hydrogel, and the mixture can subsequently be neutralized to a pH of 7.4. Furthermore, an acidic solution injection into an infarcted heart may cause a localized inflammatory response, though slow gelation time is a significant challenge during its use in cardiac tissue engineering [31,32]. To address this problem, Mirsadraee et al. produced a decellularized matrix containing collagen that is non-cytotoxic to human dermal fibroblasts [33,34]. Ott et al. have demonstrated that a decellularized rat heart may be utilized as a model by which to create a functioning heart via the introduction of smooth muscle cells (SMCs), endothelial cells (ECs), and cardiomyocytes [35]. Fibrinogen and thrombin act collectively during the hemostatic coagulation process with calcium catalysis to form fibrin gel, which is nontoxic and biodegradable. Consequently, it is a promising candidate for tissue engineering. The implantation of adult rats’ femoral arteries with hollow fibrin gel tubes filled with neonatal cardiomyocytes was reported by Birla et al. [36,37,38]. Rat cardiomyocytes were implanted in fibrin gel by Huang et al., who discovered that the cells’ contractility could be preserved for up to two months while maintaining normal pacing ability [39]. The fibrin/cardiomyocyte constructions were cultivated by Black et al. in conditions of radial contraction [40].

ii. Synthetic polymeric hydrogels

Poly(ethylene glycol) (PEG), polylactide (PLA), polylactide-glycolic acid (PLGA), polycaprolactone (PCL), polyacrylamide (PAA), and polyurethane (PU) are examples of synthetic materials utilized in cardiac tissue engineering [41,42]. Some physical and chemical characteristics, for instance modulus, water affinity, and degradation rate, can be easily tailored to fulfill the needs of cardiac muscle tissue engineering and thus serve as an excellent candidate for tissue engineering [43,44]. However, one of the main issues with synthetic polymers is their possible cytotoxicity. Some of the widely used polymers in drug deliveries are PEG, PLA, and PLGA. Indeed, it has been previously established that several other polymers, such as PAA and PU, are non-toxic both in vitro and in vivo. Ring-opening polymerization of ethylene oxide produces PEG, a water-soluble polymer. It is biocompatible and widely used in drug delivery. Many authors have reported diacrylate-modified PEG polymerized via photo-polymerization in the presence of UV light to form PEG hydrogel. PEG gel has been extensively employed by way of a supporting matrix in each aspect of tissue engineering, including the liver, pancreas, bladder, skin, and cartilage, due to low protein adsorption and inert surface. Low protein affinity, however, does not help with cell adhesion. To improve cell adhesion, techniques including conjugating proteins or peptides that are sticky to cells and adding growth factors have been employed [40,45,46]. The 3D environment of cardiomyocyte–matrix interactions has been studied using PEG hydrogel [47,48]. Poly(2-hydroxyethyl methacrylate) (PHEMA) that has pendant hydroxyl groups is a hydrophilic polymer [49]. The cardiac tissue engineering field has made use of PHEMA hydrogel in the canine epicardium as reported by Walker et al. Here, they rooted poly(ethylene terephthalate) (PET) mesh-reinforced PHEMA gel [50,51]. Because peptides/proteins mimic the amide structure ability of PAA gels, they are promising candidates for cardiac tissue engineering. As with PEG hydrogels, crosslinking is desirable when seeking to fabricate PAA hydrogels. PAA gel has very unique properties and a thermo-responsive sol–gel transition, which allows PAA solution to remain liquid at lower temperature and a soft gel at human body temperature. These properties indicate them to be innovative injectable hydrogels that can be delivered in vivo in a liquid form through a needle before they become solid gels once in contact with warm tissue [52,53]. A widely used thermosensitive polymer, Poly(N-isopropylacrylamide) (PNIPAM), has LCST value 32 °C. At room temperature, PNIPAM is liquid, turning into gel at 37 °C. These features were explored by Okano et al. to form cell sheets for tissue engineering. Neonatal cardiomyocytes were cultivated on top of tissue culture plates coated with PNIPAM at 37 °C. The culture plate was cooled to room temperature and the cells were raised to form a monolayer cell sheet. After that, the cardiomyocyte monolayers were inserted into the heart that had been infarcted. Four weeks following implantation, there was a noticeable improvement in cardiac function [54,55].

**Table 1:** Compiled literature on injectable polymeric hydrogels with various properties as well as on their application and concerns with regard to cardiac tissue engineering.

Hydrogel	Properties	Application	Concern	Ref.
Natural				
Collagen	Biocompatible, biodegradable	- Collagen scaffolds are versatile, with many relevant physical, chemical, mechanical, and morphological properties that are tailorable to achieve specific functions. - Collagen can be extracted in large quantity from a wide range of tissue sources at high purity, and at relatively low cost. - It has an abundance of potential ligand sites to promote cellular activity during myocardial tissue regeneration. - Collagen, in particular fibrillar type I, is the main constituent of the ECM of many hard and soft tissues. - It supports myocyte alignment and contributes to matrix resistance to deformation during the cardiac cycle, playing an important role in the maintenance of myocardium shape, thickness, and stiffness.	- The low stiffness of gel-like systems and poor ability to create a spatial bio-mimetic environment somewhat limits its in vivo applications. - There is difficulty in designing collagen scaffolds that have a nonlinear elasticity similar to the heart muscle and it is therefore difficult to develop a scaffold which beats synchronously with the recipient heart. - There is an unmet need for vascularization which is crucial for adequate mass transport, cell survival, electromechanical integration and functional efficiency of the transplanted cardiac patch.	[28,31,56,57,58,59]
EHT	Electricity conductive	- It can be easily shaped or cast to the complex geometry of the myocardium and so - can provide efficient bonding to the native tissue. - It can be generated easily with minimal variation. - Has similar characteristics to heart tissue, meaning that they are suitable for drug toxicology. - It can be fused together to create relatively large constructs.	- A true adult cardiomyocyte phenotype has not been reproduced. - Larger EHTs with sufficient cells for clinical relevance have not yet been produced. - As of yet, EHT viability has not been maintained as vascularization is unable to reach the core of the grafts.	[60,61,62]
Fibrin	Biocompatible, biodegradable, availability	- Fibrin gel/cardiomyocytes construct formed a mature cardiac tissue with a dense capillary network. - It possessed all normal cardiac functions, including contractility under electric stimulation and synchronous pacing with an outside electric signal.	- Slow gelation and fast degradation in vivo.	[63,64,65,66,67,68]
Matrigel	Closely resemble native structure	- Use in ECM-mimicking. - Ability of faster vascularization compared with other natural hydrogels.	- Potentially carcinogenic concerns.	[69,70,71,72]
Alginate	Biocompatible, biodegradable, non-toxic, non-immunogenic, non-thrombogenic	- Cell transplantation, drug, and protein delivery and wound healing. - Can be directly and locally injected into the infarcted myocardium or via intracoronary injection and therefore its use does not require open surgery.	- Mammals lack the alginase enzyme; however, the partial oxidation of alginate chains promotes degradation under physiological conditions. - Alginate hydrogels have poor bioresorbability and low cell adhesiveness, which may lead to adverse tissue interaction and poor wound-healing properties.	[73,74,75,76,77,78]
PHA	Biocompatible, bioresorbable	- Ideal for soft tissue engineering. - Potential to maintain their structural integrity over a longer period due to the surface degradation vs. bulk degradation observed in PLA and PLGA. - Diverse mechanical properties. - Used for different aspects of cardiac tissue engineering such as patches and valves. - Three-dimensionally-printed bespoke structures, electrospun fiber sheets, gyrospun fiber sheets, porous 3D scaffolds, melt-extruded and dip-molded tubular structures, solvent cast films, hydrogels, microspheres, and nanospheres.	- The medical grade PHA production method is mostly quite expensive and not many commercial sources are available. - Often, different PHAs require blending together in order to produce a material with suitable mechanical properties for cardiac applications. - Some PHAs are susceptible to thermal degradation.	[79,80,81,82,83]
Silk	Bioresorbable	- A variety of silk-based biomaterials have been approved by the FDA in drug delivery. - Good adherence to native cardiac tissue. - Causes little to no immunological response. - Silk-based biomaterials have diverse tuneability.	- Silk usually has to be combined with other materials in order to make it suitable for cardiac applications. - The natural production of silk by spiders leads to batch-to-batch variability between different species and even within individual spiders.	[84,85,86,87,88,89,90,91]
Chitosan	Biocompatible	- Can be processed into films, membranes, hydrogels, fibers, scaffolds, and sponges. - Can be used for drug delivery. - Useful in applications such as myocardial patches.	- Has a rigid crystalline structure, making it difficult to dissolve in common solvents. - Chitosan is derived from individual organisms leading to batch-to-batch variability.	[92,93,94,95,96,97,98,99,100]
Synthetic				
Poly ethylene glycol (PEG)	Bio-inert, biocompatible, FDA approved for drug delivery.	- Can be used for drug delivery. - Has diverse tuneability.	- Low cell adhesion, concerns of non-injectable and toxic nature of small crosslinkers, not degradable.	[101,102,103,104,105,106,107,108,109]
Poly(2-hydroxyethyl methacrylate) (PHEMA)	Biocompatible and available for functionalization.	- Controlled thickness and stiffness. - Diverse mechanical properties.	- Modulus mismatch. - Non-degradable.	[110,111,112,113]
Polyamides	Biocompatible, bioresorbable.	- Fast gelation time, injectability. - Versatile for chemical modification.	- Pure polyamides are not degradable. - Non-elastic.	[31,114]
Poly(N-isopropylacrylamide) (PNIPAM)	Biocompatible, non-toxic.	- Used in several in vivo studies for neonatal rat ventricular cardiomyocytes.	- Gelation at 37 °C and cannot be used below that temperature.	[115,116,117,118,119,120,121]
PEG-dimethacrylate (PEGDMA)	Biocompatible, Biodegradable.	- Used in in vitro for muscle fiber formation.	- Lengthy and low reproducible synthesis can make difference in batch-to-batch production.	[122]

## 3. Properties of Injectable Hydrogels

### 3.1. Self-Healing Mechanism in Hydrogels

**Figure 2:**
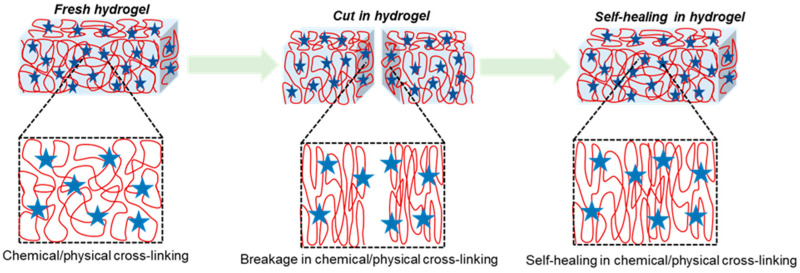
Hydrogel formation via chemical or physical cross-linking between polymers, showing self-healing efficiency where cuts in hydrogel resulted in the breakage of chemical or physical cross-linking bonds, which enable self-healing via reforming bonds where red globular shows polymer chain and blue star represents cross-linking agents.

The unique properties of polymeric hydrogels are self-healing, a process which is driven from reversible chemical or physical linkages. The conductivity, fast adhesion, and stimulus responsiveness are intrinsic properties of self-healing hydrogels. More significantly, there should be sufficient mechanical toughness in the biological use of such hydrogels. The healing capacity of hydrogels influenced by a physical bond is demonstrable on reversible non-covalent interactions such as ionic or electrostatic interactions, hydrophobic interactions, host–guest bond interactions, and hydrogen bonds [123,124,125]. The hydrogel cross-linking bonding method is depicted in Figure 2. Zhang et al. described 36% *w*/*v* PVA-based hydrogels with excellent self-healing features. It is mainly hydroxyl groups in the polymer matrix that encourage hydrogen bond restructuring and enable the material to be self-healable [126]. Rumon et al. stated that graphene-oxide-functionalized polyacrylamide gel resulted in 70% healing due to richness in the -OH and -COOH groups, which experience physical bond formation with the polymer chain [127].

Mechanically strengthened polymeric gels can be made using irreversible covalent bonds. Additionally, the self-healing feature can be introduced via reversible crosslinked covalent bonds in dynamic chemistry, where a damaged or cracked polymeric chain network can be restored via reversible covalent bonds, for example, a disulfide bond, dynamic imine bond, or Diels–Alder reaction [54,128,129,130]. Self-healing hydrogels based on a dynamic imine bond have been reported by Zhang et al. to be used in biological applications [131]. The pH-dependent self-healing hydrogel was invented by Guo et al. Its self-healing efficacy is highly reliant on the pH of the solution, and it must reach a certain degree of healing efficiency in order to be employed in biomedical applications [132].

### 3.2. Gelation Time

**Figure 3:**
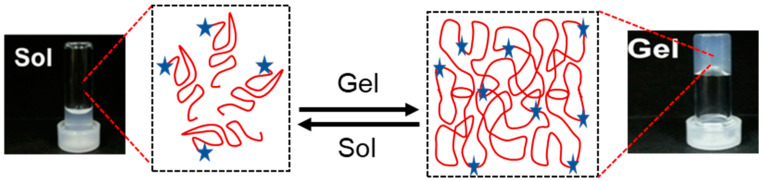
Gelation time of hydrogel showing sol and gel quick transition in presence of stimuli red globular shows polymer chain and blue star represents cross-linking agents, sol vials show fluid liquid and gel vial shows soft gel formed.

For cardiac tissue engineering applications, injectable hydrogels should ideally undergo a quick phase transition from a liquid solution (when in a catheter before injection) to a gel state (after being injected into the targeted location) [133] as shown in Figure 3. The best possible cellular engraftment, hydrogel deployment, and retention of cells and/or biomolecules should all be achieved throughout the sol–gel transition [134,135]. In order to do this, an injectable hydrogel’s gelation kinetics should keep the substance in solution while it is still inside the catheter and then quickly, possibly in a matter of seconds, form a gel upon injection into the intended tissue [136]. Natural injectable hydrogels frequently exhibit delayed sol-to-gel transition rates (15 min to 24 h), which, when injected into cardiac tissue with a high concentration of vessels, may exacerbate the loss of cells and biomolecules because the semiliquid gel can be washed away. Slow gelation rates raise the possibility of disrupting normal blood flow and ultimately causing tissue necrosis, in addition to worries about the loss of therapeutic molecules and cells. Conversely, the gelation periods of synthetic injectable materials are usually substantially rapid, ranging from a few minutes to a few seconds [137].

### 3.3. Gelation Stimuli

**Figure 4:**
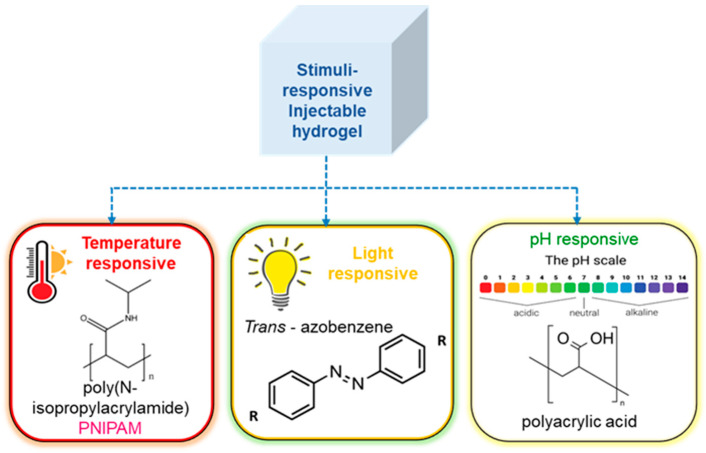
Stimuli responsive gelation of hydrogel, showing the effect of various stimuli such as temperature, pH, light.

Stimuli that are triggered on gelation include light, pH, temperature, and others shown in Figure 4 [138]. Thermoresponsive hydrogels have the advantage of not requiring UV light for crosslinking, which might cause endogenous oxidative damage to DNA, or other potentially irritating solutions, which could be less damaging to encapsulated cells. The formation of thermoresponsive hydrogels can occur through a variety of processes, including swelling behavior brought on by temperature changes. Because of its affinity for water, the hydrogel holds water and swells below its lower critical solution temperature (LCST). The hydrogel becomes increasingly hydrophobic above the LCST, and the swelling process terminates by forming a stable hydrogel. Triblock copolymers are composed of a hydrophilic–hydrophobic–hydrophilic backbone that undergoes sol-to-gel transition based on micelle formation due to an increase in temperature [139,140,141,142]. Injectable hydrogels can also go through the sol-to-gel transition by another well-liked gelation method called light-induced crosslinking or photopolymerization. A monomer’s polymerization or a hydrogel’s crosslinking can both be aided by light [143,144,145]. The use of pH as a trigger to cause hydrogel gelation has also been investigated [146,147,148]. Alimirzaei et al. reported on these pH-responsive hydrogels, developing a pH-sensitive chitosan hydrogel for the encapsulation of human adipose MSCs (hADSCs). When this hydrogel approaches physiological pH, a sol–gel transition occurs. These novel hydrogels represent a promising candidate, with suitable mechanical properties for cardiac tissue engineering [149].

### 3.4. Mechanical Strength

Post myocardial infarction, alterations in the myocardial biomechanical microenvironment and cellular loss may result in unfavorable ventricular remodeling, such as ventricular dilatation and wall stress, which can ultimately cause myocardial dysfunction, fibrosis, and hypertrophy [150,151]. The myocardial experiences irreversible necrosis as a result of a series of events that damage the cell membrane and disturb the structure of the heart tissue, which sets off this maladaptive remodeling. In a perfect world, injectable hydrogels would provide the damaged myocardium with appropriate mechanical support to make up for the tissue damage. Even though a lot of injectable materials—like ECM hydrogels—have relatively soft mechanical properties that make it simple to inject them into a damaged myocardium, they are usually not strong enough to provide the injured cardiac tissue—which is constantly under strain and contraction—with sustained, continuous mechanical support [152,153]. Researchers have discovered that the mechanical strength of injectable hydrogels may be changed to resemble the mechanical strengths present in heart tissue by adjusting the quantity of polymer dissolved in aqueous solutions. An injectable PEG hydrogel with mechanical tailoring was created by Chow et al. who created and examined hydrogels with polymer concentrations of 5, 10, 20, and 30% *w*/*v*. The materials’ respective shear moduli were 0.8, 6.9, 17.2, and 35 kPa. The 10% and 20% *w*/*v* hydrogels were found to most nearly resemble the shear moduli of the infarcted (18 kPa) and normal (6 kPa) myocardium, respectively. They observed that, 10 weeks after injecting the 10% hydrogels into an MI rat model, the rats’ pathogenic ventricular remodeling was reduced in comparison with the controls [149,154].

### 3.5. Biocompatibility

Biocompatibility is a property of any medical material that is used in contact with a living body. The requirements for hydrogels are nontoxicity, an ability to be sterilized, and biocompatibility. Cytotoxicity assesses how the hydrogel affects the viability of cells. Assays like MTT, XTT, or live/dead staining can be used for this [155,156,157]. The breakdown products of biodegradable hydrogel must be non-toxic and readily absorbed or eliminated by the body. The loss of the protective microenvironment that would have supported the injured cardiac tissue, the time it takes for the encapsulated cells to become established and engrafted, and the therapeutic molecules that are locally required for their action, can all be problematic for matrices that degrade too quickly and may be cleared away soon after injection. The MMP family of proteases causes the majority of natural materials, including collagen, fibrin, and decellularized extracellular matrix, to degrade rapidly [158,159,160,161]. Wassenaar et al. introduced doxycycline, an MMP inhibitor, into the ECM in order to encourage comparatively slow degradation of the decellularized porcine ventricular myocardium. Two distinct techniques were used to do this, one included chemically cross-linking doxycycline to the hydrogel, and the other involved combining doxycycline with the hydrogel matrix in solution during production [162].

## 4. Hydrogels Functionalization

**Figure 5:**
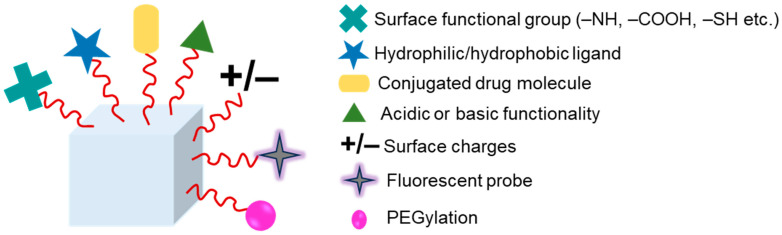
Hydrogel functionalization via chemical linkages such as attaching surface functional group, hydrophilic/hydrophobic ligand, drug conjugation, acid or base functionality, introducing surface charges, fluorescent probe, PEGylation.

Grafting or functionalization of polymers is required to boost hydrogels’ mechanical strength. Hydrophobic or hydrophilic compounds can covalently link to various functional groups to modify hydrogels. This gives them intriguing qualities for use in biological applications [163]. Polymeric hydrogels can have their physical characteristics and solution behavior tuned by chemical modification, such as adding hydrophobic, acidic, basic, or other functionality to the polymer chain. Hydrogels have been chemically modified using a variety of techniques and can be used for specific purposes. Important examples of chemical modification of polymeric hydrogels include PEGylation, grafting of polymeric chains by polymerization, and formation of polymer–drug conjugates [164]. Figure 5 shows a schematic illustration of the hydrogels’ surface functionalization, with some examples of polymers and their cross-linking agents for various application in Table 2.

A robust approach for enhancing and boosting compatibility is grafting. Grafting is such a simple and efficient way to change the chemical composition of polymers. Many natural polymers, including cellulose, hyaluronic acid, starch, and chitosan, have been employed in grafting to enhance performance. By reacting with functional groups or polymerizing a monomer, a hydrophilic or hydrophobic polymeric moiety can be grafted on the polymeric chain’s backbone. This process is known as “grafting through”, “grafting on”, and “grafting from”. The former strategy has been explored the most among these [165,166]. Enzymatic synthesis or microwave grafting are two methods that are widely available. In grafting, the microwave irradiation method shortens the reaction time and uses less hazardous solvents. As a result, when compared with polymeric hydrogels grafted using the conventional approach, the hydrogels grafted using microwave technology have better qualities [167,168].

**Table 2:** Examples of functionalized hydrogels and their cross-linking agents, showcasing their diverse applications in cardiovascular tissue engineering.

Polymer	Cross-Linking Agent	Cross-Linking Type	Application	Ref.
Surface functionalization				
PVA	NH_4_OH, NaOH, CH_3_COOH	Thermal	Cardiac	[169]
Hydrophobic/hydrophilic ligand functionalization				
Dextran	Dextran bifunctionalized with methacrylate and aldehyde	Photo	Vascular	[170,171]
Conjugated drug molecule				
PEG, PVA	Cyclodextrin, doxorubicin	Host-Guest	Heart valve	[172,173]
Acidic and Basic functionality				
Gelatin	Hydroxyphenylpropionic acid	Photo-enzymatic	epicardium	[174]
Chondroitin sulfate	Furfuryl amine grafted chondroitin sulfate	Diels-Alder	myocardium	[175]
Surface charge				
Chitosan	lecithin	Thermal	Cardiac blood vessel	[176]
Fluorescence probe				
PNIPAM/Gelatin	PNIPAM-based copolymer, Thiol dye modified gelatin	Thermal and Michael addition	Cardiac patch	[177]
PEGylation				
PEG	Norbornene-terminated PEG	Michael addition	Vascular tissue engineering	[178]

## 5. Hydrogels in Cardiovascular Tissue Engineering and Biomedical Application

**Figure 6:**
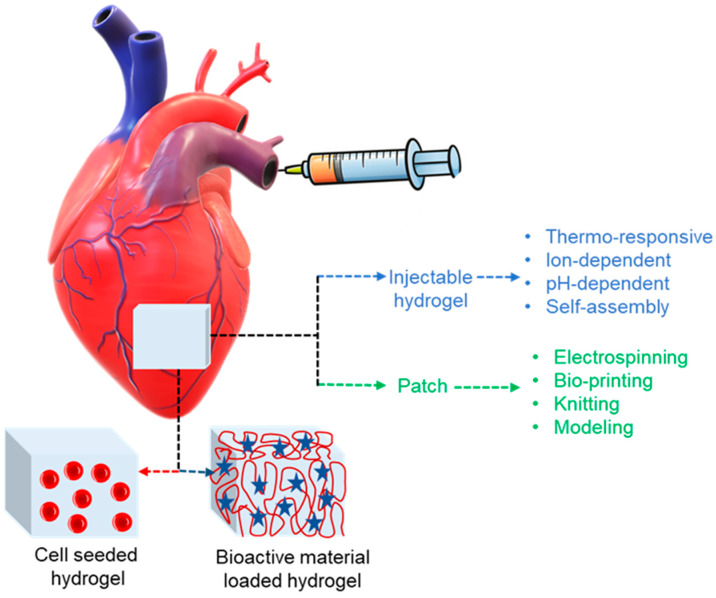
Polymeric materials in cardiac applications with two major treatment approaches for heart injury. These are injectable hydrogels and cardiac patches functionalized with bioactive materials like RNA, small molecules, proteins, or they can be seeded with cells where red buttons represents cell, red globular indicate polymer chain and blue star shows bioactive material.

The multidisciplinary zone of tissue engineering practices ideas from biology and engineering to produce biological tissue replacements that preserve, enhance, or restore function. Scaffolds are essential for polymers and tissue engineering. Natural and synthetic polymers are valuable resources for scaffolding. A characteristic approach is to immobilize cells in a 3D hydrogel. Numerous materials that are categorized based on gelation mechanism can be taken into consideration, based on the desirable gelation time, cell type, damage site, and desired period of disintegration along with their preclinical investigations [179,180]. Wang et al. used injections of brown adipose tissue-derived stem cells that had been fixed in a thermoresponsive chitosan gel to confirm cardiomyocyte differentiation and improve cell survival. Using appropriate catheters, in situ formation of hydrogels can be made with the benefit of less invasive delivery. With this method, catheter occlusion from early gelation must be prevented, and the hydrogel should develop as soon as the damaged area is injected [181]. Bencherif et al. provided a technique by which to create a preformulated hydrogel with shape memory effect that is still injectable in order to get over this issue. The foundation material in this case was alginate, which was metharcylated to enable subsequent radical polymerization. The preformulated alginate hydrogel, which is nanoporous and polymerized at −20 °C, may be inserted using a needle or catheter. When the shear force exerted is released, the hydrogel reverts to its initial shape [182]. Hydrogels can be loaded with bioactive substances or seeded with cells for use in the heart in order to promote stem cell migration or differentiation and, consequently, tissue regeneration, as shown in Figure 6. Though the process of developing injectable cell-loaded hydrogels yielded encouraging results, the number of cells that successfully differentiated into cardiomyocytes in these experiments was inadequate [14]. However, from a regulatory perspective, a cell-free approach is quite appealing, and, since it is not patient-specific, it can be produced in an off-the-shelf, up-scalable manner. Current in vitro and in vivo research has concentrated on the utilization of hydrogels that release drugs bio-actively. Bio-active compounds that are now in use and encapsulated include tiny molecules like RNA, prostaglandins, growth hormones, and other factors like bone and sonic hedgehog and bone morphogenetic protein-2 [183,184,185,186].

The flawless porous hydrogel scaffold is mechanically robust, biocompatible, biodegrades at a pace that does not release any toxins, has a surface shape that encourages cell interaction, serves as a template for the tissue, and promotes cell attachment, proliferation, and differentiation. The extracellular matrix (ECM) is an ordered network made up of different proteins and polysaccharides that gives cells structural support. Therefore, materials based on proteins and polysaccharides may have the benefit of promoting cell adhesion and activity [182,187]. Hydrogel composition has an influence on in vivo performance when building hydrogels for cardiac applications, where mechanical strength and stiffness are critical design factors. For instance, MI causes wall weakening, necessitating the use of a hydrogel to mechanically maintain the injured area. It should be remembered that excessively stiff hydrogels may result in diastolic dysfunctions. As a result, it is crucial to define suitable mechanical qualities. Furthermore, it is well known that all cells, including stem cells, react to their surroundings, and it has been demonstrated that hydrogel stiffness is a key factor in stem cell differentiation [188].

While injectable hydrogels are the subject of much research right now, there are alternative biomaterials-based approaches that can help with heart healing. Solid prefabricated scaffolds, often referred to as patches, can be placed epicardially into the injured heart, for instance. These scaffolds may be loaded with bioactive chemicals or seeded with cells, just like hydrogels, as shown in Figure 6. Not only may patches transfer cells or release bioactive compounds for medicinal purposes, but they can also lower dilation and offer mechanical support. However, in designing a cardiac patch, the following specifications must be taken into account: the patch should be strong mechanically and flexible at the same time. However, materials that are overly stiff might cause diastolic dysfunctions and result in non-contractile structures. Most of the time, it is intended that the patch degrades after sufficient remodeling [138,188].

Because gelatin is digested or denatured collagen, it has been extensively studied and used for the generation of widely applied semi-synthetic compounds that are extensively used, including gelatin methacryloyl and various biorthogonal derivatives. Despite missing biological binding motifs, alginate-based natural hydrogel derived from algae has been used because of its facile polymerization controlled by cations like Ca or Mg and because its adaptable mechanical characteristics. Hydrogels made from silk and its derivatives have also been created by adding electrically active particles or chemical groups that may be photo crosslinked. In order to determine if the added nanoparticles will encourage the development of myocardial tissue, researchers also used alginate-based cardiac patches with magnetically responsive nanoparticles. The patches were subjected to an external magnetic stimulation at a physiologically relevant frequency (5 Hz) [189,190,191].

Electrically conductive hydrogels have attracted much interest lately in the biomedical domain for tissue engineering and bioelectrode applications. Conductive hydrogels are often created by combining conductive elements with hydrophilic polymers, as traditional hydrogels are electrically insulating. Therefore, it is possible to construct conductive hydrogels that have mechanical and electrical characteristics that are comparable to those of healthy cardiac tissue. Electrically conductive biomaterials can stimulate the development of CMs in the infarcted heart and stop it from progressing to arrhythmia. They can also improve electrical coupling and cardiac contraction/relaxation functions. As a result, conductive hydrogels may be a useful tool for post-MI cardiac tissue healing by providing mechanical support for heart tissues and efficient electrical signal transmission throughout the myocardial. To create conductive hydrogels for cardiac tissue engineering, a variety of electrically conductive materials, such as conductive metals, polymers, and nanomaterials have been employed. Pre-polymers containing conductive materials can be crosslinked for in situ reactions, including photo-crosslinking, click chemistry, Schiff base reactions, and Michael reactions, in order to create injectable conductive hydrogels following intramyocardial or intrapericardial injection. Through in situ polymerization of tetraaniline-polyethylene glycol (TA-PEG) and thiolated hyaluronic acid (HA-SH) via Michael addition, an injectable conductive hydrogel was created by Wang et al. The infarcted heart was injected with adipose-derived stem cells (ADSCs) and lipofectamine nanocomplexes carrying plasmid DNA-eNOs (endothelial nitric oxide synthase) put into the gels. Electrocardiography, cardiography, and histological investigation revealed that they had improved structural and functional cardiac function along with elevated proangiogenic factors and enhanced expression of eNOs in the myocardium. Another intriguing use of conductive hydrogels for stimulating heart tissue regeneration is in conductive cardiac patches. A cardiac patch does not need invasion of the myocardial or pericardium, as a contrast with injection into the myocardium. Patches made of conductive hydrogel can offer a 2D electrical bypass around the epicardium. The common methods for attaching cardiac patches, including conductive hydrogels, to the epicardium include light irradiation, staples, or traditional surgical sutures. These methods may result in bleeding, occlusion of the blood supply, or increased inflammatory reactions [13,192,193].

Injectable hydrogels have the important role of stimulating angiogenesis, an essential component of heart regeneration. Hydrogel injections reduce pathologic remodeling and stimulate neoangiogenesis in the superficial myocardium. Using injectable hydrogels to create vascularized hearts and vascular analogs has various benefits. Furthermore, because of their networked porosity, hydrogels allow blood vessels to open and encourage vascularization in vivo. Hydrogels’ biological characteristics include their capacity to bind to and interact with cells, which aids in the vascularization processes. An injectable hydrogel not only thickens the ventricular wall but also improves the geometry of the left ventricle, supports cardiac tissue, and encourages the regeneration of cardiac tissue. In the interim, an injectable hydrogel may alleviate left ventricular dilatation and infarct expansion by lowering the pressure on the ventricular wall. Additionally, an injectable hydrogel has high safety and therapeutic efficacy and considerably improves heart function. It has been demonstrated that the recovery of cardiac function is associated with improvements in cardiomyocyte survival, repair, and myocardial pathological remodeling. The injectable hydrogel can save at-risk cardiac cells by delivering proangiogenic agents that promote angiogenesis, enhance local neovascularization, reduce inflammatory response, and rescue the ischemic myocardium by improving and optimizing the microenvironment in the damaged heart tissue. Overall, hydrogels have the potential to open up new avenues for the creation of vascular grafts and nanocarriers, which might improve the efficacy of the pharmacological therapy and revascularization strategies that are discussed here [29,193,194].

### 5.1. Hydrogel-Based Therapy

Collagen deposition is, from a pathology perspective, the final stage of remodeling following MI. If the hypotension in the myocardium is not alleviated, the ventricles will continue to dilate, ultimately leading to CHF. It has been suggested that injecting a supportive gel into the infarcted heart to relieve the high wall stress will stop this process. Ventricular dilation has been found to be attenuated by injection of PNIPAM-based copolymers. This shows that, by giving the infarcted region enough mechanical support, hydrogel-only treatment may be useful in postponing the cascade that results in CHF. Unfortunately, this treatment is merely passive and is unable to regenerate new myocardial due to a scarcity of cells [183,184,185,186].

### 5.2. Stem-Cell-Loaded Injectable Hydrogel-Based Delivery

Hydrogel-based cell therapy is a potential approach by which to create heart tissue. During injection, the viscous hydrogel keeps the cells in the intended location and the weakening heart wall is mechanically supported by the gel itself. The supplied cells may be able to survive and develop into cardiomyocytes in the gel environment in order to rebuild heart muscle in the interim. Hydrogels and biochemicals can be co-delivered to promote cell growth, survival, and differentiation. Adipose-derived stromal cells (ASCs), cardiomyocytes (CMs), epithelial cells (ECs), cardiac progenitor cells (CPCs), endothelial progenitor cells (EPCs), embryonic stem cells (ESCs), induced pluripotent stem cells (iPSCs), and bone marrow-derived mesenchymal stem cells (BMMSCs) are just a few of the stem cells that have been the subject of research in recent years. Furthermore, there are several obstacles in the way of stem cells’ clinical use for heart regeneration because of their extremely low survival and retention rates in ischemic tissue. Stem-cell loaded injectable hydrogel offer stem cells the perfect milieu to preserve their stemness, which encourages their survival and development. It has been discovered that MSCs can help treat MI therapeutically. Alginate hydrogels have been shown in one study to improve MSC retention and impulse conduction in swine MI models. According to a different study, type I collagen and a new injectable thermosensitive hydrogel consisting of copolymerized N-isopropylacrylamide/acrylic acid/2-hydroxylethyl methacrylate-poly(e-caprolactone) might be used to retain MSCs. In the MI models, it has been demonstrated that this hydrogel greatly increases the transplanted MSCs’ survival, stimulates neovascularization, attenuates fibrosis, and further improves heart function [13,138,189,190,192].

### 5.3. Drug-Loaded Injectable Hydrogel-Based Delivery

Therapeutic drug-loaded injectable hydrogel can be injected to the human or animal body to improve safety and efficacy at the targeted tissue at a controlled rate and time. Hydrogels, with their moderate pore size for improved drug delivery and protection of pharmaceuticals against degradation in the body, have demonstrated considerable benefits over traditional approaches for attaining the required concentration of a medication in the body. They can also shorten the time it takes for the medicine to reach its target tissues and help with its sustainable release. Because of their biocompatibility and biodegradability, alginate, chitosan, cellulose, dextran, PLA, and PLGA can be used as potential candidates. The characteristics of polymers, target drugs and target tissues determine which hydrogel and drug is best for tissue engineering [163,164,165,166].

### 5.4. Cytokines, Nucleic Acids, Plasmids and Peptides

MI have been treated with a variety of cytokines. Growth factors, such as fibroblast growth factor-2 (FGF-2), hepatocyte growth factor (HGF), insulin-like growth factor 1 (IGF-1), nerve growth factor (NGF), platelet-derived growth factor (PDGF), transforming growth factor beta-1 (TGF-b1), and vascular endothelial growth factor 165 (VEGF165), are most frequently used to control cell fate and function during regeneration. However, rather than a burst release profile, the distribution of growth factors necessitates a moderate release pace. Hydrogels play a vital role in the release of growth factors, as they are able to maintain control over the release time. Furthermore, these substances have the capacity to improve growth factor stability and specificity. The majority of the research has shown intriguing findings that indicate that different hydrogels can provide an ideal platform for growth factors to release and facilitate the restoration of cardiac function after MI. All of these growth factors have already been applied to various hydrogels in the investigation of MI models. Therapeutic targets like small nucleic acids and plasmids can be conjugated to various hydrogels and have been used to release siRNA against MMP2. Following MI, MMP2 is in charge of cardiac remodeling. Hydrogels are broken down by proteases, releasing active siRNA that further reduced MMP2 in primary CFs. After four weeks in a rat model of MI, hydrogels releasing siMMP2 significantly increased ejection fraction (EF), stroke volume (SV), and cardiac output (CO) in comparison with hydrogels containing control siRNA. They also reduced myocardial thickness in the infarct area. Peptides can be delivered via hydrogels to keep heart function from declining in MI models. A chitosan–collagen-based hydrogel immobilized with pro-survival angiopoietin-1-derived peptide was reported to better reduce post-MI cardiac remodeling by injection into the peri-infarct/MI zone in rat models with MI than gel without peptide or gel with PBS groups. After two weeks, this hydrogel began to break down around the third week. Both scar thickness and cardiac dysfunction were markedly improved by the combination. Furthermore, after applying hydrogel, more CMs were found in the MI zone without triggering an inflammatory reaction [165,166,167,168,179].

### 5.5. Oxygen Delivery System

Following MI, cardiomyocytes are susceptible to cell death because of a deficiency of oxygen in the surrounding cardiac tissue. One possible strategy to preserve the ischemic heart cells and encourage heart healing is to introduce controlled oxygen release into the infarcted region. Because of the reduced blood flow in the ischemic region, current oxygen delivery systems are unable to adequately distribute oxygen into the infarcted area. Using hydrogels with quick gelation times and thermosensitive properties in combination with oxygen-releasing microspheres, some researchers have created a novel oxygen delivery method. It has been demonstrated to selectively diffuse to infarcted heart tissue and release oxygen on a regular basis. With 1% O_2_, the concentration of infarcted heart tissue, this system’s ability to provide oxygen continuously for four weeks greatly increased the survival of cardiac cells under ischemic circumstances. Following four weeks of this system’s treatment to infarcted hearts, there was a considerable drop in the number of macrophages and an improvement in cell survival. Additionally, myofibroblast density and collagen deposition were noticeably decreased, while angiogenesis was markedly enhanced, all of which contributed to a considerable improvement in heart function [13,192,193,194].

## 6. Three-Dimensional and Four-Dimensional Bioprinting as Recent Technology

**Figure 7:**
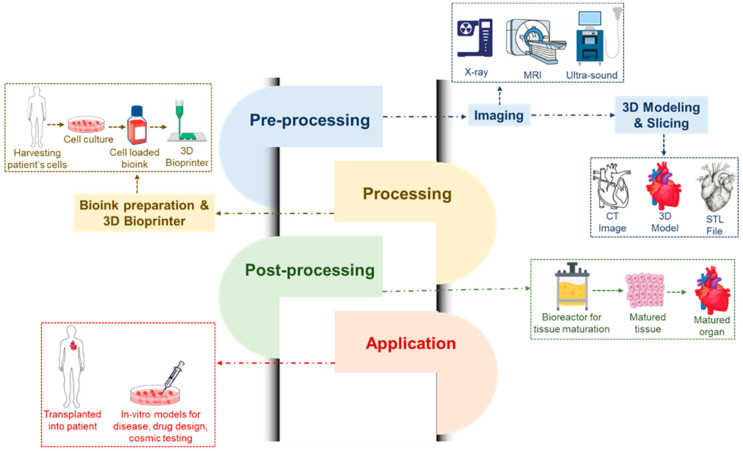
Three-dimensional and four-dimensional bioprinting, showing process analysis where pre-processing techniques required imaging via X-ray, MRI and ultrasound followed by three-dimensional modeling and slicing with CT imaging, three-dimensional modeling and STL file capturing. The next step is processing data by bio-ink preparation and 3D printing, where steps are followed by harvesting the patient’s cell, collecting cell culture, putting them into bio-ink and finally printing them using a 3D printer. Afterwards, post-processing requires reaction in a bioreactor for collected cell tissue growth, the mature tissues are then collected and inserted into a mature organ. Finally, a 3D-printed materials are applied either in in vitro models development, drug design and cosmic testing or transplant into patient’s body.

For tissue engineering and regenerative medicine, three-dimensional (3D) printing is a new and exciting area. It has the ability to create 3D organs or tissues for in vivo practice. Moreover, the drug delivery effectiveness and safety may be evaluated using 3D-printed structures. Under the exact management of pre-manufactured computer software, the bio-ink used in 3D printing may change from a liquid phase into a solid form to produce functioning 3D architectures. The bio-ink comprises a variety of cell types and biomaterials [38,39,180]. Several cell types, such as endothelial cells, cardiomyocytes, fibroblast cells, mesenchymal cells, and smooth muscle cells, can be used for 3D bioprinting in heart regeneration. Three-dimensional bioprinting can also function as the heart’s natural ECM. An ideal material for bioprinting is easily tunable, has a specific viscosity, is able to change from a liquid to a solid state, be nontoxic, have good biocompatibility without triggering an immune response or inflaming the body, have the right mechanical qualities, be biodegradable, and have the right pore size [193,194]. For 3D bioprinting, biomaterials like alginate, agarose, chitosan, collagen, HA, and decellularized ECM can be employed. A more advanced printing technology is 4D bioprinting, which is far more sophisticated than 3D bioprinting. Four-dimensional bioprinting offers a dynamic means of generating structural and cell changes over time to build tissue architectures that mimic those seen in nature. Seeding 4D-bioprinted tissue into the required shape, for example, a tube that resembles a blood vessel, is one method of creating the 4D architecture. Stimulating tissues to aid in their self-assembly is another method [195,196,197,198,199]. In the future, this 4D bioprinting method will enable more innovative research and it shows promise for heart regeneration. Figure 7 shows the overall process engineering regarding use of hydrogels in cardiac tissue engineering.

Here, Table 3 shows the compiled literature regarding the process evolution for printing and fabrication using several injectable hydrogels. Some old techniques have several disadvantages, which result in the need to invent smart techniques to overcome them. Further research and clinical trials are ongoing for the development and scaleup of novel techniques which are cost-effective and useful for cardiac tissue engineering.

## 7. Challenges in Hydrogels Usage

Injectable hydrogels are gaining rapid attention in ECM analogs as a viable method of encapsulating and releasing various therapeutic drugs or cells. These hydrogels may also be used as functional cardiac tissue engineering tools, providing a therapeutic response for the replacement of damaged heart tissue. However, a few important problems remain to be addressed, including when to inject, how the injectable hydrogel materials biodegrade, and certain critical hydrogel characteristics for myocardial repair. Hydrogels biodegrade in the heart for a sufficient amount of time to continue serving the intended therapeutic purpose in the infected zones. In contrast, a shorter time frame is required to ensure that the material fully degrades before the disease site fully recovers and does not have any side effects [194,211]. More significantly, the simultaneous change in the hydrogel’s stiffness as a result of degradation may not coincide precisely with the myocardium’s time-dependent change in stiffness following myocardial infarction. The intended material must be able to offer continuous mechanical support in order to facilitate the progressive transfer of the cardiac load from an unhealthy tissue to a newly developed one, in order to solve this crucial issue. In light of the foregoing assumption, the hydrogel systems ought to be engineered to maintain their integrity in vivo for a minimum of one week, given that the majority of cell death transpires during the initial days following MI and should ultimately undergo total degradation after around six weeks. The hydrogel should be effectually injected as soon as MI occurs. This will give the myocardium mechanical support and allow for tissue healing, which is likely to be hindered by delayed injection after MI. As a result, the most often used injection timing in an animal model is right after MI; however, as patients typically receive therapy at least a few hours after MI, this method is not realistic in a clinical setting [212,213]. The standards used to assess the effectiveness of injectable hydrogel-based cardiac regeneration are not uniform. It is still unknown from currently conducted short-term studies in small animals which of these parameters represent the most important one for the patients’ lifespan, despite the fact that all of the well-documented typical parameters, such as wall thickness, left ventricular volume, shortening fraction, ejection fraction, and vascularization of the infarct region, are essential for myocardial regeneration [56,214,215]. From a translational perspective, some injectable hydrogels have a remarkable track record, but they also face several obstacles. These include the potential for complex synthesis procedures that are challenging to scale up, high costs, and toxicity to human tissue. Despite the high clinical esteem for PEG and PVA, carrier systems based on these pre-polymers are difficult to scale up due to their high cost and the cross-linkers necessary to prepare PVA hydrogels. Several hydrogels encounter obstacles on their journey to the clinic, and some of them fade away over time, without ever reaching a patient. Because of this, the majority of hydrogel carriers that have received clinical approval are based on old concepts that have been redesigned with a new idea. To determine the importance of these factors for effective myocardial regeneration and the patient’s lifetime, further research in a large animal model with longer-term follow-up monitoring is required. Furthermore, it is important to evaluate the injected hydrogel’s overall biosafety.

## 8. Conclusions and Future Perception

This review summarizes the state of the art in cardiac tissue engineering using hydrogels. The formulation of innovative functional biomaterials that can aid in the healing of damaged heart tissue following cardiovascular illnesses is essential for the efficient treatment of cardiovascular diseases nowadays. There is a lot of potential for using hydrogels in heart regeneration and repair following MI. The bioactivity, biocompatibility, and biodegradability of hydrogels means that it can serve as a potential candidate for the same. Because of their strong binding affinity, swelling ability, and absorption capacity, hydrogels are the perfect biomaterials for agent delivery systems and cardiac implants. The cross-linked spatial structure of hydrogels can be used to increase colonization resistance, prolonging their half-life in vivo and delaying fast breakdown. In the meantime, hydrogels can elevate electric conductivity benefits towards contraction. Hydrogels are utilized to give therapeutic medicines with controlled release while also giving structural support to an infected area. Hydrogels and encapsulated bioactive compounds work well together meaning that, as a consequence, both must operate at the same time for the benefit of heart regeneration. Clinical studies and commercial products are few in the field of heart regeneration medicine, so it is necessary to look into clinical viability further. In cardiovascular tissue engineering, injectable hydrogels and hydrogel-based cardiac patches are the most often utilized applications. While hydrogel-based cardiac patches decrease MI stress, which passively reduces ventricular expansion. Injectable hydrogels allow for the in-situ delivery of bioactive chemicals and cells to support in situ repair and regeneration. Cardiac patches made of hydrogel function as mechanically supporting biomaterials. The benefits of intelligent hydrogels increase the range of applications, improve the techniques of regulation, and improve the injectable hydrogels’ and hydrogel-based cardiac patches’ controllability. Nutrient transfer can be aided by the three/four-dimensional (3D/4D) network structure of hydrogels; however, diffusion is the primary method used to do this. In terms of the building of scaffolds that closely resemble native heart tissue, multi-material structures made using 3D/4D printing techniques and customized structures for individual patients have great potential for new, near-future advancements in cardiac tissue engineering.

In conclusion, hydrogels have a lot of potential for heart regeneration and repair following MI. Intelligent hydrogels and patches extend the spectrum of applications in cardiac tissue engineering by rearranging their structure via physiochemical changes in the surrounding medium. In cardiac tissue engineering, there are possibilities and problems that coexist and so it is necessary to further look into clinical viability. In the near future, hydrogels should provide enormous translational promise for heart regeneration and repair. Recent years have seen remarkable advancements in the field of cardiac tissue engineering, with hydrogels serving as scaffolding for medicinal compounds. Therefore, one of the main areas of focus for materials science and tissue engineering research in the upcoming decades will be the creation of innovative injectable hydrogels that may serve as soft scaffolds for medicinal chemicals.

## Figures and Tables

**Table 3:** An overview of the benefits and drawbacks of printing and fabrication techniques for cardiac tissue engineering.

Fabrication Techniques			Advantages	Disadvantages	Ref.
No true architecture	Mold casting	Mold casting	- Simplicity and cost - High cell survival - Range of compatible materials - Mechanical properties - Scalable feasibility	- Low-cost architectural control - Limited thickness	[200]
	Pore forming	Solvent casting, Particle leaching, cryogelation	- Control of pore size - No specialized equipment cost	- Use of organic solvents - Limited scaffold thickness - Mechanical properties - Time consuming leaching - Limited pore architecture	[200]
	Electrically produced	Solution electrospinning	- Nanometer features - Range of compatible materials - Scalable feasibility	- Use of organic solvents - Limited scaffold thickness - Produced high stiffness substrates	[201]
	Textile based	Weaving, Braiding, Knitting	- Simple - Scalable feasibility	- Specialized equipment cost - Not widespread - Limited porosity	[202]
True architecture control	Build and seed	Stereolithography	- High architectural control - Self-supporting process	- Only photo stimuli polymer - Remove supporting materials - Use of UV light - Toxicity of photo-initiator - Specialized equipment cost	[203]
		Selective laser sintering	- No solvent required - Self-supporting process - High architectural control - Range of compatible material	- High temperature - Material in powder form - Rough surface - Special equipment cost	[204]
		Melt electrospinning writing	- No solvent required - Control over pore size, porosity, fiber diameter - High architectural control - Range of compatible material	- Limited thickness - Range of available material	[205]
		Fused deposition modeling	- Speed of printing - Good reproducibility	- Restricted materials with good melt viscosity - High temperature - Filament requirement - Limited resolution	[206]
	Bioprinting	Laser guided direct writing, Laser induced forward transfer, Bio-laser printing, 3D/4D bioprinting	- Range of cell and biomaterial - Precise cell printing - Single cell resolution - High cell viability - Process at room temperature	- Low cell viability - Time consuming - Limited 3D structure - Weak structural support - Special high-cost instrument - Multi-material printing expense	[207,208,209,210]
